# Transdiagnostic treatment of emotional disorders in people with multiple sclerosis: randomized controlled trial

**DOI:** 10.1186/s40359-020-00480-8

**Published:** 2020-10-31

**Authors:** Nabi Nazari, Masood Sadeghi, Ezatolah Ghadampour, Davod Mirzaeefar

**Affiliations:** Department of Psychology, Faculty of Human Sciences, Lorestan University, Khorramabad, Iran

**Keywords:** Unified protocol, Emotion regulation, Transdiagnostic, Depression, Anxiety trial registration authority

## Abstract

**Background:**

Multiple sclerosis (MS) is a neurodegenerative disease of the central nervous system. MS is significantly associated with a high rate of psychological, behavioral, and emotional consequences. Despite the frequent mental disorders, high rate of psychological comorbidities, and emotional problems in people with MS (PwMS), these conditions are often underdiagnosed and undertreated. This study aimed to examine the efficacy of a group format of the unified protocol for the transdiagnostic treatment of emotional disorders in adult PwMS associated with an emotional disorder.

**Methods:**

Seventy adult PwMS were randomized using an internet-based computer system to either the unified protocol (*n* = 35) or treatment as usual condition. The assessment protocol included semi-structured clinical interviews and self-reports evaluating diagnostic criteria, depression, anxiety and worry symptoms, emotional dysregulation, and affectivity.

**Results:**

The parametric test of analysis of covariance, followed the intent to treat analyses, revealed the unified protocol significantly changed depression symptoms (Cohen’s *d* = 1.9), anxiety symptoms (Cohen’s *d* = 2.16), worry symptoms (Cohen’s *d* = 1.27), emotion dysregulation (Cohen’s *d* = 0.44), positive affect (Cohen’s *d* = 1.51), and negative affect (Cohen’s *d* = 1.89) compared with the control group. The unified protocol also significantly improved outcome scores at the end of treatment relative to baseline (*p* < .001).

**Conclusion:**

The findings support that the unified protocol could be an additional efficient psychological treatment for PwMS.

*Trial registration* IRCT, number: IRCT20190711044173N1. Registered 31october 2019, https://en.irct.ir/user/trial/40779/view.

## Background

Multiple sclerosis (MS) is a chronic, progressive, neurodegenerative disease of the central nervous system. MS is significantly associated with psychological, behavioral, and emotional consequences [1]. For people with MS (PwMS), the risk of being affected by an emotional disorder (such as depression, anxiety, anger, euphoria) is higher than healthy populations and other chronic conditions [2]. Depression, experienced by up to 50% of PwMS, can negatively impact functioning, disability, pharmacological therapy adherence, and suicidal ideation [3, 4]. In addition to specific-disorder, psychological comorbidity is common in PWMS and is correlated with a greater disability over time [5]. Suicidal behaviors in PwMS are two times higher than the general population [6]. Besides depression, maladaptive coping strategy and emotional dysregulation were the most potent predictors that have predictive accuracy for suicidal ideation as many as 85% [7]. This neurological disease that affects the limbic system will induce emotional disturbances. Also, comorbidity has an additive adverse effect on patients’ mental quality of life and is associated with an increased risk of debilitating complications, further increasing disease burden. For instance, during the MS, risk-related behaviors may expose an individual to various problematic environmental agents [8]. Despite the frequent mental disorders, comorbidities, and emotional problems in PwMS, these conditions are often underdiagnosed and undertreated [9].

Several reasons for this underdiagnosed condition have been documented. In a neurologic setting, evidence highlights the weakness of the DSM criteria application [10]. Also, the MS syndrome’s heterogeneous nature and the potential for confusing specific somatic complaints of MS (e.g., fatigue) with depression symptoms may lead to falsely elevated underdiagnoses rates. Moreover, disorder-specific interventions and treatments based on primary and secondary diagnoses are not suggested to be effective with complex cases [11]. Furthermore, Disorder-specific protocols can be difficult to justify when the clinical reality is complex, and comorbidities are the norm, particularly in chronic somatic disease (e.g., MS) [12].

Cognitive Behavioral Therapy (CBT) programs have demonstrated effectiveness in promoting mental health in PwMS for treating depression [13]. Nevertheless, effective treatments for anxiety are lacking [14]. Recent findings have shown that CBT was less efficient than other interventions in the psychological treatment of PwMS [15]. Transdiagnostic and integrated therapies have emerged as recommended approaches for the treatment of several co-occurring mental health disorders, as they provide a more parsimonious [16] and more efficient strategy to working with comorbid presentations [17]. Some studies have suggested that a transdiagnostic treatment approach for PwMS can be appropriate [18].

Transdiagnostic approaches refer to identifying the etiology and maintenance mechanisms that are common in multiple disorders [19]. In emotional disorders, neuroticism has been considered a key etiology mechanism shared by all emotional disorders [20]. Other mechanisms identified have been rumination, suppression, anxiety sensitivity, and misappraisal [21], frequently reported in PwMS [22]. These mechanisms can increase or maintain persistent negative emotions and may affect physical and psychological functioning. From this perspective, transdiagnostic treatment consists of techniques that serve to target an identified set of underlying core processes [19]. Emotion regulation seems to play a critical role in the treatment of complex cases, diagnoses with a combination of psychological risk factors, or comorbidities [23]. PwMS experience higher rates of negative emotions related to different situations such as support family members, body image, pregnancy worry, uncertainty about the relationship, and sexual dysfunction [24, 25].

The Unified Protocol is a CBT transdiagnostic emotion-focused skill-based therapy [26, 27]. The unified protocol has been manualized to be applied to the treatment of anxiety disorders, depression, and other emotional disorders in which emotion dysregulation is a core component [28]. The protocol has been adopted in 12 to 14 sessions in a group format [29]. Numerous studies have supported the efficacy of the unified protocol in improvements on anxiety and depression symptoms, functional impairment, and well-being [27, 30] chronic diseases [31], and social, job, and general performance [32].

### Current study

The unified protocol is equally effective as gold‐standard specific disorder protocols for individuals with comorbid emotional disorders [27]. Regarding the prevalence of emotional disorders, high comorbidities, frequent emotional problems, and the high prevalence of the risky-behaviors during the MS [8], the application of unified protocol, as an emotion-focused, skill-based intervention, could be beneficial through targeting emotion regulation mechanism, improvement emotional disorder comorbid conditions, and temperamental changes concerning neuroticism. However, there is a lack of empirical evidence on the unified protocol and MS. The purpose of the study was to examine and develop the efficacy of a group format of the unified protocol for adult PwMS with depression or anxiety symptoms. We hypothesized that at post-intervention, treatment group participants would show significant improvements in measure of depression, measures of anxiety and worry, the measure of emotion regulation, and the measure of affectivity relative to the treatment-as-usual (TAU) group. Also, we hypothesized that treatment group participants would demonstrate significantly improve on dependent variable scores compared with baseline at post-intervention.

## Methods

### Participants

**Fig. 1:**
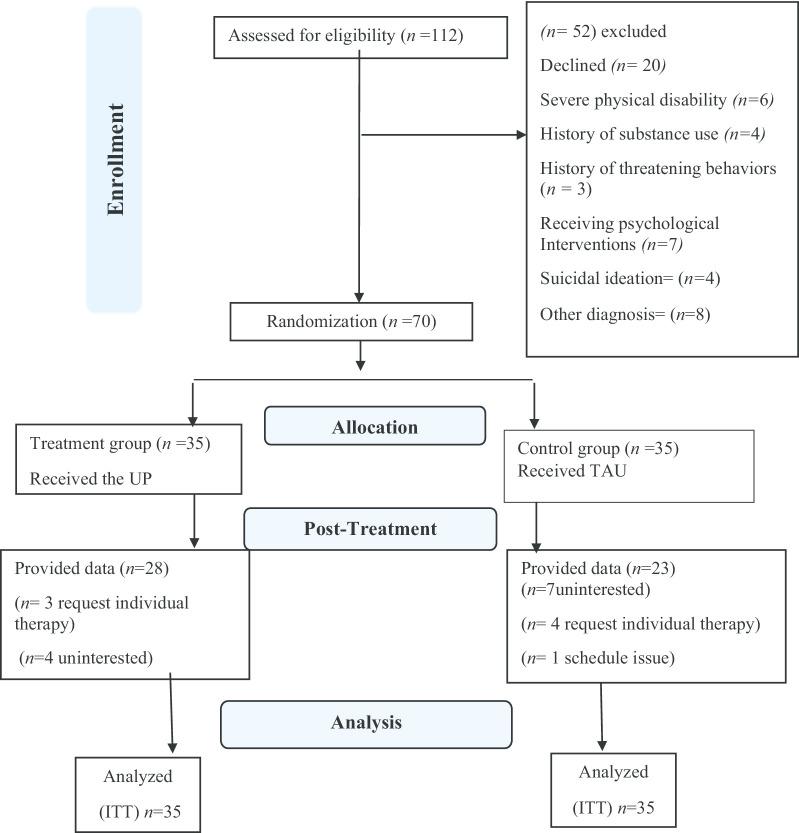
The participants flow chart diagram. *UP* unified protocol, *TAU* treatment as usual, *ITT* intent to treat, *n* frequency

The consort diagram is illustrated in Fig. 1. Participant recruitment efforts included notifying MS clinics and MS associations through the use of brochures and posters. A total of 122 people were assessed to participate in the trial. Of these, 70 (44 females) met all eligibility criteria. Participants’ ages ranged from 22 to 47 years. The mean age of the participants was 35.30 (*SD* = 3.01). The participants were generally well-educated. The baseline assessment is demonstrated in Table 1.

**Table 1:** Demographic characteristics of the sample (N = 70)

Item Characteristic	Value	Test	*p*
MS duration*, n* (%)			
to 3 years	39 (28.2)	χ^2^ = 0.92	*.*396
3 to 7 years	31 (44.75)		
Gender, *n* (%)			
Women	43 (63.4)	χ^2^ = 3.65	*.*056
Man	27 (38.6)		
Education, *n* (%)			
Primary education	12 (17.1)	χ^2^ = 30.22	< .001
Higher education	58 (82.9)		
Marital*, n* (%)			
Single	28 (40)	χ^2^ = 2.80	.091
In relationship	42 (60)		
Principal diagnosis SCID			
Depressive disorder,* n* (%)			
MDD	34 (48.6)		
Dysthymia	5 (7.1)		
Anxiety disorder, *n* (%)			
GAD	22 (31.4)		
SAD	9 (12.9)		
Continues variables			
Age, *M* (SD), y	35.13 (5.28)	*t*(68) = 1.01	.321
MS duration, *M* (SD), y	3.31 (1.37)	*t*(68) = 1.03	.304
PANAS-PA, *M* ((SD)	26.00 (3.75)	*t*(68) = 1.55	.122
PANAS-NA, *M* ((SD)	27.42 (2.72)	*t*(68) = − 0.6	.148
PSWQ, *M* (SD)	47.55 (9.46)	*t*(68) = − 1.23	.221
HADS-A, *M* (SD)	12.43 (1.37)	*t*(68) = − 1.89	.059
HADS-D, *M* ((SD)	12.84 (1.63)	*t*(68) = − 1.24	.214
DERS, *M* ((SD)	110.14 (12.17)	*t*(68) = − 1.50	.132

*n* frequency, *y* years, *SCID-I–IV* structured clinical interview for *DSM-IV* axis i disorders, *MDD* major depressive disorder, *GAD* generalized anxiety disorder, *SAD* social anxiety disorder, *t* independent *t* test, *SD* standard deviation, *PANAS-PA* positive and negative affect schedule-positive affect, *PANAS-NA* positive and negative affect schedule-negative affect, *HADS-A* the hospital anxiety and depression scale-Anxiety, *HADS-D* the hospital anxiety and depression scale-depression, *PSWQ* Penn State Worry Questionnaire, *DERS* difficulties in emotion regulation scale

*Inclusion criteria included* (a) fluent in Persian, (b) at least 18 years of age, (c) a valid diagnosis of MS, (d) obtained a consort, (e) received a diagnosis of depression or anxiety disorders based on *DSM–IV* [33] [Diagnostic and Statistical Manual of Mental Disorders, fourth edition; American Psychiatric Association, 2000], (f) medical agreement or valid referral document for participation.

*Exclusion criteria included* (a) present or history diagnosis of schizophrenia, psychosis, or organic mental disorder, (b) other chronic physical illnesses, (c) pregnancy or Breast-feeding, (d) risk or history of threatening behaviors, (e) missed three consecutive sessions (f) receiving psychological or psychiatric treatments during the study (e.g., antidepressant or anxiolytic medication), (g) moderate to high cognitive impairment or physical disabilities.

### Measures

*Structured Clinical Interview for DSM-IV Axis I Disorders* [SCID I–IV: 34, 35] was used in the current study. The diagnosis was moderate to good (Kappa coefficient higher than (0.6). Most interviewees and interviewers reported the desirable implementation of the local version of SCID-I. Kappa was higher than 0.4 for all the diagnoses except for Generalized Anxiety Disorders. The Kappa was above 0.85 in most of the diagnoses, and in half, it was above 0.9, indicating acceptable reliability [36]. (Note: at the time of the study, a Persian version of the SCID for DSM-V was not yet validated. The clinical psychologists and supervisors on the study created a cross-referenced checklist and determined that patients met criteria for anxiety or depression based on both the DSM-IV and DSM-V.)

#### Primary outcomes measures

*The hospital anxiety and depression scale* [HADS: 37]. The HADS is a highly reliable screening measure for assessing anxiety and depression in PwMS. The HADS consists of 14-items, two sub-scales, 7-items for anxiety (HADS-A), and seven items for depression (HADS-D). A suggested cutoff score of 11 demonstrated high sensitivity (90%) and specificity (92%) for the Anxiety subscale and high sensitivity (77%) and specificity (81%) for the Depression subscale [38]. This scale demonstrated acceptable reliability in this study (*α* = 0.90).

*Difficulties in Emotion Regulation Scale* [DERS: 39]. The DERS is a 36-item, self-report questionnaire that measures overall difficulties in emotion regulation. The DERS consists of six subscales: (1) no acceptance of emotional responses, (2) difficulties engaging in goal-directed behavior, (3) impulse control difficulties, (4) the lack of emotional awareness, (5) limited access, and (6) lack of emotional clarity. Respondents rated their emotional state on 1(almost never) to 5 (almost always). The total score range of 36–180. A recent study has found that a DERS total score above 97 identified a clinical sample [40]. DERS has high internal consistency (α = 0.93). Internal consistency in the current study was acceptable (*α* = 0.92).

#### Secondary outcomes

*The Positive and Negative Affect Schedule* [PANAS: 41]. The PANAS is a brief self-report scale that determines positive and negative affects with two independent ten descriptors. The PANAS demonstrates the two core dimensions of mood positive affect (PA) and negative mood affect (NA). Each item is rated on a five-point scale with a range from very slightly (1) to extremely (5), indicating the extent that the participant has experienced that feeling over the past month. The PANAS has shown highly internally consistent, largely uncorrelated positive affect (0.89) to negative affect (0.95), whereas the discriminant correlations are quite low [41]. Internal consistency in the current study was acceptable (*α* = 0.80).

*Penn State Worry Questionnaire* [PSWQ: 42]. The PSWQ is a 16-item self-report measure that determines an individual’s tendency to worry and intensity and excessiveness of worry on a scale of 1 (not at all typical of me) to 5 (very typical of me). The PSWQ has demonstrated reliable psychometric properties, suitable internal consistency, and test–retest reliability in the local MS population. This measure is suggested for transdiagnostic approach assessments. Internal consistency in the current study was acceptable (α = 0.83).

### Procedure

The study was a single-blind, parallel randomized controlled trial comparing psychological intervention group, based on the unified protocol, with a TAU control group. The study, including all assessments and treatments, was conducted at the MS Clinic, located within the MS Centre. The study’s methods and procedures were reviewed and approved by the Institutional Human Research Ethics Committee and the National Institute for Medical Research and Development, prospectively. “We used the CONSORT checklist and The TIDieR checklist when writing our report (see Additional file 1).”

First, interested PwMS were notified about the study’s goals, benefits, and risks, session numbers, randomization, and group allocation chance through telephone or face-to-face interviews. Only who gave verbal informed consent to participate in the study were asked to present their physician agreement or refer to the study participation. The neurologists and clinical psychologist evaluated physician agreements, referrals, medical documents for recent medication prescriptions, and examined the subjects. The eligibility criteria are related to medical conditions obtained; the participants completed the assessment protocol. Individuals who met the SCID-I–IV criteria for depression or anxiety disorders were requested to sign the consent form. All participants obtained a signed written consent form.

At last, only consented subjects who received a valid depression or anxiety diagnosis were selected for randomization. The outcomes were assessed at two time-points: Time 1: pretreatment to pre-allocation includes baseline, Time 2: immediate after intervention: post-treatment assessment.

### Sample size

The sample size for Analysis of covariance (ANCOVA) was conducted using G*Power 3.1 analysis [43]. A priori power analysis was conducted, using an alpha of 0.05, a power of 0.8, and medium to large effect size (Cohen’s *f* = 0.35) to determine the sample size. According to G*Power, the desired total sample size was 64. Therefore, 70 participants recruited, allowing for a 10% loss of data (dropping out prior treatment, end treatment assessment).

### Randomization and blinding procedures

Randomization was performed using a computer-generated sequence (www.randomizer.org). A list of anonymous participant identification numbers was used to randomly allocate participants to treatment or control without any restrictions. An independent statistician performed new randomization after each 10-participants allocated. The concealed was disclosed at the end of the study. The independent statistician carried out the randomization and informed the patients and the monitoring board about the allocation. To masking condition assisting, participants were instructed not to disclose any information about the intervention and diagnostic status. Psychological evaluators, data collectors, assessors, and statistic investigators were blinded to the intervention, participants’ group, and pervious diagnostic status.

### Interventions

#### The unified protocol intervention

The program and sessions were structured based on the latest comprehensive published manual developed by Barlow and colleagues [26–28]. Group therapy consists of 12 weekly 2-h sessions. The treatment content is included topics about Motivation, psychoeducation, mindfulness, cognitive flexibility, emotion-driven behavior, and emotional avoidance, interoceptive exposure (IE), in vivo exposure, and relapse prevention. The summary of each module content and intervention schedule is demonstrated in Table 2. (See Additional file 2 for the more detailed description.)

**Table 2:** Content and the number of sessions for module

Module	Schedule	Content and the number of sessions for module
One	Week 1	Setting goals and maintaining motivation (1 session)
Two	Week 2	Understanding emotions (1sessions)
Three	Week 3and 4^a^	Mindful emotion awareness (2 sessions)
Four	Week 5 and 6	Cognitive flexibility (2 sessions)
Five	Week 7	Countering emotional behaviors (1sessions)
Six	Week 8	Understanding and confronting physical sensations (1session)
Seven	Weeks 9 to 13^a^	Emotion exposures (5 sessions)
Eight	Week 14	Recognizing accomplishments and looking to the future (1 session)

The Modules of three, four, five, six, and seven are Core modules

^a^Week 3 and week 13 can be deleted based on the 12 session program

#### Treatment-as-usual intervention

The control group received the TAU that consists of 12 weekly two hour sessions. The program included psychoeducation and life-long MS considerations (4 sessions), sharing experiences (4 sessions), and marital and parental counseling (4 sessions). This treatment could be considered as a psychoeducation intervention delivered in routine care focused on reducing negative emotions.

### Risk

Routine medical and psychological evaluations were accomplished before all activities (e.g., assessments, interviews, and treatment sessions). Regarding safety, the medical health care staff included two physicians and four experienced nurses also alerted in case of emergency conditions during all activities. The follow-up phase coincided with a viral epidemic. Therefore, in order to ensure the safety of participants, no follow-up was performed.

### Statistical analysis

All analyses were conducted with SPSS software version 25 (version 25, SPSS Inc., Chicago, IL), two-tailed with an alpha level of 0.05 to determine statistical significance, following an Intention-to-Treat (ITT) analysis approach. With the ITT approach, study participants are analyzed as members of the treatment group to which they were randomized regardless of their adherence to, or whether they received, the intended treatment. Given that the analysis was based on ITT principles, the data for all randomized 70 individuals were included in the final report. To handle missing data, the last provided data (the last observation-carried-forward (LOCF) were considered as a next point for dropping data. An independent t-test was conducted to explore whether the participant was equivalent at baseline (Time 1).

The parametric test of analysis of covariance (ANCOVA) was conducted to compare the effectiveness of the unified protocol intervention and TAU at post-treatment (Time 2). The scores on the baseline are treated as a covariate to control for pre-existing differences between the groups. Preliminary checks were conducted to ensure no violation of the assumptions of normality, linearity, homogeneity of variances, and homogeneity of regression slopes. Levene’s test was used to determine normality and homogeneity of variance. Also, the homogeneity of regression slopes assumption was tested. A paired *t* test was conducted for all measures between (Time1–Time2) to investigate within groups' changes. The within-group effect size was calculated for both groups.

Effect sizes are reported as partial eta squared. Standardized effect size Cohen’s *d* was calculated for pre-post treatment changes based on means and standard deviations [44]. Effect size estimates were interpreted conservatively, with 0.2, 0.5, 0.8, reflecting small, medium, and large treatment effects, respectively.

## Results

### Descriptive characteristics at baseline

There were no significant differences in terms of demographic features, age, duration, education, and other dependent variables at baseline (see Table 1). There was no significant difference between participants with depressive disorders and participants with anxiety disorders in the study (χ^2^ = 0.91, *p* = 0.33). The unified protocol participants’ ages ranged from 25 to 44 years, with a mean of 33.06 years (*SD* = 6.22). The TAU participants’ ages ranged from 22 to 47 years, with a mean of 33.89 years (*SD* = 5.24). Seven (20%) from the unified protocol group left the experiment before Time 2. On average, participants had a very high degree of adherence and protocol well tolerated; 28(80%) of the unified protocol group completed the treatment sessions and completed all the post-treatment measures. Also, 12(37%) of the TAU group dropped out at post-treatment. Finally, 50 (71%) of all participants completed the study.

A one-way between groups ANCOVA was conducted to assess the impact of the unified protocol of reported all measures. The ANCOVA assumptions were examined before submitting the test results. Homogeneity of Variance was tested using Levene’s test, indicating insignificance of *p* value (*p* > 0.05). The Homogeneity of Variance assumption was met for: HADS-A [*F*(1, 68) = 3.46, *p* = .07), for HADS-D [*F*(1, 68) = 3.61,* p* = .06] for DERS* F*(1, 68) = 1.89, *p* = .17, for PANAS-PA [*F* (1, 68) = 1.51, *p* = .38], for PANAS-NA [*F* (1, 68) = 2.80, *p* = .10], and for PSWQ *F*(1,68) = 1.14, *p* = .28]. Also, the homogeneity of regression slopes assumption was met for: HADS-A [ *F*(1, 66) = 1.02, *p* = .32), for HADS-D [*F*(1, 66) = 1.56,* p* = .14] for DERS* F*(1, 66) = 1.895,* p* = .17, for PANAS-PA [*F* (1, 66) = 0.04, *p* = .84], for PANAS-NA [*F* (1, 66) = .065, *p* = .80], and for PSWQ [*F*(1, 66) = 2.10, *p* = .15]. There was no significant interaction between the covariates and the intervention.

### Treatment results

*Depression treatment results* The ANCOVA was conducted on HADS-D. The results showed a significant main effect for group, *F*(1, 67) = 74.91, *p* < .001, η^2^*p* = .52, and Cohen’s *d* = 1.9. For the group, the unified protocol significantly less HADS-D scores than TAU. The adjusted post-treatment mean for the unified protocol group (*M* = 8.01) was significantly less than that for the TAU group (*M* = 12.79).

*Anxiety treatment results* The ANCOVA results showed a significant main effect for group, *F*(1, 67) = 82.47, *p* < .001, η^2^*p* = .55, and a Cohen’s *d* = 2.1. For groups, the unified protocol significantly less HADS-A scores than TAU. The adjusted post-treatment mean for the unified protocol group (*M* = 8.08) was significantly less than that for the TAU group (*M* = 11.68).

*Emotion dysregulation treatment results *The ANCOVA was conducted on DERS. The results showed a significant main effect for group, *F*(1, 67) = 11.04, *p* = .001, η^2^*p* = .14, and a Cohen’s *d* = 0.42. For the group, the unified protocol significantly lower DERS scores than TAU. The adjusted post-treatment mean for the unified protocol group (*M* = 97.52) was significantly less than that for the TAU group (*M* = 112.81).

*Affectivity treatment results* The ANCOVA was conducted on PANAS-PA and PANAS-NA. The PANAS-NA. The results showed a significant main effect for group, *F*(1, 67) = 62.19, *p* < .001, η^2^*p* = .48, and a Cohen’s *d* = 1.89. The adjusted post-treatment mean for the unified protocol group (*M* = 18.98) was significantly less than that for the TAU group (*M* = 27.50).

The PANAS-PA results showed a significant main effect for group, *F*(1, 67) = 47.99 *p* < .001, η^2^*p* = .42, and a Cohen’s *d* = 1.51. The adjusted post-treatment mean for the unified protocol group (*M* = 30.68.) was significantly higher than that for the TAU group (*M* = 24.83).

*Worry treatment results* The ANCOVA was conducted on PSWQ. The results showed a significant main effect for group, *F*(1, 67) = 34.78, *p* < .001, η^2^*p* = .34, and a Cohen’s *d* = 1.2. The adjusted post-treatment mean for the unified protocol group (*M* = 34.24) was significantly lower than that for the TAU group (*M* = 46.26).

A Paired *t* test was carried out to examine treatment effectiveness between Time 2 and Time1. These findings revealed the unified protocol had a significant effect on symptom improvement (see Table 3). Means and standard deviations were calculated at Time1 and Time 2 (see Table 4). There were no adverse events associated with this trial. A comparison test Between Time 2 and Time 1 revealed no significant differences for the TAU group.

**Table 3:** Paired *t* test and within group effect size at post-intervention

Item	Treatment as usual group		Unified protocol group		
	*t(34)*	*p*	*t(34)*	*p*	*Cohens’d* [95%CI]
Anxiety	1.95	.062	10.54	*p* < .001	− 2.78 [− 3.67, − 2.01]
Depression	− .46	.643	7.83	*p* < .001	− 2.01 [− 2.78, − 1.35]
Emotion difficulties	− 2.01	.052	3.07	*p* < .01	− 0.20 [− 0.66, − 0.14]
Positive affect	1.98	.055	− 7.01	*p* < .001	1.44 [0.86, 2.08]
Negative affect	− .98	.327	8.26	*p* < .001	− 1.95 [− 2.65, − 1.31]
Worry	.65	.512	6.89	*p* < .001	− 1.45 [− 2.02, − 0.92]

*Time2–Time1 *post-treatment to baseline, *CI* confidence interval of the difference

**Table 4:** Mean (SD) at pre-treatment at pre-treatment and post-treatment

Item	Treatment as usual group		Unified protocol group	
	Time1	Time2	Time1	Time2
Anxiety	12.40 (1.37)	11.69 (1.65)	12.57 (1.40)	8.09 (1.82)
Depression	12.60 (1.65)	12.80 (2.00)	12.88 (1.80)	7.9 (2.79)
Emotion difficulties	108.74 (8.46)	114.3 (16.63)	110.1 (12.17)	106.5 (20.42)
Positive affect	26.43 (3.98)	25.00 (3.40)	25.20 (3.36)	30.51 (3.79)
Negative affect	26.69 (3.03)	27.51 (3.77)	27.43 (3.27)	18.97 (5.04)
Worry	47.00 (8.42)	46.09 (8.60)	48.00 (8.86)	34.43 (9.40)

Time1: pre-treatment, Time2*:* post-treatment to

The SCID-I -IV demonstrated 21 of 35 participants in the unified protocol group (60%) no longer met the diagnostic criteria for their principal diagnosis at the end of the study. The SCID-I–IV demonstrated no worse condition for all participants at Time2.

## Discussion

MS is associated with a broad array of emotional disorders, negative symptoms, social interference, and physical disability that compromise well-being [45]. This study aimed to examine the efficacy of a group format of the unified protocol for the transdiagnostic treatment of emotional disorders and symptoms in adult PwMS. Our approach was based on the key development of the emotion regulation mechanism outlined in the unified protocol transdiagnostic treatment framework for emotional disorders. The results revealed that PwMS, who participated in the unified protocol intervention group, demonstrated significant improvements in depressive and anxiety and worry emotion regulation, and affectivity outcomes at post-treatment compared with those who participated in the TAU group. Our findings revealed significant changes in depression measure, in anxiety measure, and in worry, in emotion regulation measure, and affectivity measure in the unified protocol group at post-treatment relative to baseline.

The results are consistent with studies that indicate the unified protocol is effective in improving emotional disorders [46–48]. The core modules of the unified protocol are relevant to depression. Briefly, negative affect (e.g., sadness, shame, anger) and maladaptive, avoidant reactions to negative affect are targeted in the unified protocol. For example, in emotional disorders, worrying is a critical maladaptive cognitive process contributing to the maintenance of the disorder, and worrying can be effectively targeted by promoting adaptive emotion regulation strategies [49]. PwMS focus on the disease consequences, which may be concluded to catastrophizing future, over-estimate threat, and under-estimate their abilities to cope. Present-Focused Emotion, a core module in the unified protocol, helps the patients recognize their thoughts and feelings, and concentrate on the current condition demands, making emotional experiences feel more under control and manageable. The improvement of emotion regulation can be associated with an improvement in depression and anxiety symptoms [50].

Findings revealed significant changes in DERS at post-treatment regarding with TAU group. This study develops the unified protocol benefits on difficulties emotion regulation scale have improved other clinical outcomes [51]. Also, the results provide supports for the application of emotion regulation in promoting adaptive emotion regulation among people with mental disorders [52]. In line with our investigation, numerous researches have replicated the emotional regulation implication in the treatment of depression [53], anxiety disorders [54], excessive worry, and psychological stress [21].

A large Cohen’s *d* in the negative and positive affect was found with a higher significant effect on negative affect than positive affect. These results are the same that previous RCT, applying unified protocol in emotional disorders samples that have found changes in neuroticism/negative affect after unified protocol intervention [55]. Some studies have also found differences in extraversion/positive affect [56]. The reduction in neuroticism scores confirms the unified protocol’s theory, an emotion regulation intervention targeting specifically neuroticism/negative affect [20], a psychopathology mechanism associated with the etiology of the emotional disorders [57]. These results suggest unified protocol, which typically focuses on reductions in negative affect, may also improve positive affect. Positive affect is a malleable construct and can be influenced by unified protocol. Change in positive affect can be potentially associated with improved both psychological and clinical outcomes [58]. One possible reason could be that unified protocol helps patients understand the relevant function of all range of emotions, including “positive emotions” such as happiness, joy, pride. Another possibility could be related to the group format delivery. Group therapy facilitates the normalization of MS-related experiences, and sharing with similar people reduces the stigma associated with psychological treatment.

The current study could develop the unified protocol as a transdiagnostic approach, consisting of five core modules and practical techniques for addressing different aspects of emotion regulation. Emotion dysregulation predicts quality of life, independently of disease severity and cognitive functioning [59]. Moreover, Emotional distress associated with maladaptive coping strategies is led to poor well-being rather than disease duration or severity [60]. For example, Emotional problems among mothers with MS negatively associate with the mother’s ability to cope with the disease and positively associate with depressive symptoms in their healthy partners [61]. The unified protocol components, such as Psychoeducational courses, emotional skills, and stress self-management techniques can be beneficial to enhance well-being in MS [62]. Awareness of thoughts, beliefs, and interactions facilitates coping in PwMS [63]. Interoceptive exposure is another component of the unified protocol. PwMS are more sensitive to visceral sensations than healthy individuals [64]. Dysfunctions in interoceptive inference could underlie a range of pathologies such as anxiety [65]. In PwMS, bodily sensations are usually associated with high anxiety. Interoceptive exposure may be beneficial and facilitate a controlled coping behavior, and less stress react, gradually [66]. Also, individuals with better interoceptive perception demonstrate greater self-regulatory ability in experimental social interaction [67]. Meta-analytic evidence supports the use of the mindfulness-based intervention in PwMS to improve fatigue [68].

The study revealed surprising findings. There was no significant difference between the participants who received a depressive disorder diagnosis and those who received an anxiety disorder diagnosis at baseline. This finding is contrary to current insight and epidemiologic data in PwMS [69]. This finding is critical because anxiety receives far less attention in MS. DERS scores are related to both depression and anxiety levels in the MS sample [59]. According to DERS mean score at baseline, difficulties with emotion regulation is very high in PwMS.

We investigated the unified protocol’s feasibility in a group format to an MS transdiagnostic sample with emotion regulation problems. According to evidence considering that emotion dysregulation is connected with less willingness to participate in psychological trials [70], we classified the sample as challenging to treat. According to the treatment retention rate in this study (71%), the treatment was well tolerated. Also, the results are in line with the data provided previous trials [29, 71] confirm a significant improvement of patients treated in a group format.

## Limitations

The results from this trial should be interpreted in the context of several limitations. First, the participants were generally well-educated, which can be enhanced their abilities to gain more the unified protocol and diminish the results’ generalizability. A priori power analysis was conducted, using an alpha of 0.05. However, for three primary outcomes variables (HADS-A, HADS-D, and DERS) an alpha of 0.0167 should have been considered. The next limitation was that the no-follow-up made it difficult to assess prevention effects. One strength point of this study was the SCID-I–IV application at enrollment and post-treatment.

## Conclusion

Overall the findings provide the support that the unified protocol could be an additional efficient as a parsimonious, transdiagnostic treatment of emotional disorders for adult PwMS. Although the results are promising, more research should be conducted to extend the findings obtained in this study. Transdiagnostic research has the potential to represent better the clinical and scientific reality of mental health problems, reflecting the complexity and comorbidity that is the norm in clinical practice. The unified protocol is equally effective as gold‐standard specific disorder protocols in people without MS. Future trials are required to investigate the unified protocol effectiveness, compared with gold‐standard specific disorder protocols in the PwMS. Further studies are required to assess the cost-effectiveness and efficacy of the unified protocol intervention with larger samples to promote it as part of routine care for PwMS. Economic evaluations can be simultaneously incorporated in future trials, as this has not yet been formally evaluated. There would be value in adding qualitative components into future trials to establish the unified protocol interventions' acceptability for both clinicians and clients. The findings developed the unified protocol as a mechanistically transdiagnostic approach might be applied across multiple disorders. In a unified approach, thoughts, behaviors, feelings, and body sensations are interacting dynamically, and emotional experiences influence each of them. Unified protocol Transdiagnostic intervention facilitates PwMS to learn how to respond to their unpleasant emotions more adaptively. Given the negative consequences of anxiety and depression in PwMS, interventions such as this may reduce the risk of these adverse outcomes and produce benefits for PwMS.

## Supplementary information

**Additional file 1**. Consort.

**Additional file 2**. Treatment Protocol.

## Data Availability

The data that support the findings of this study are available on request from the corresponding author.

## Abbreviations

ANCOVA: Analysis of covariance
CBT: Cognitive Behavioral Therapy
DERS: Difficulties in Emotion Regulation Scale
DSM: Diagnostic and Statistical Manual of Mental Disorders
HADS: The hospital anxiety and depression scale
ITT: Intention-to-Treat
LOCF: Last observation-carried-forward
MS: Multiple sclerosis
PANAS: Positive and negative affect scale
PSWQ: Penn State Worry Questionnaire
PwMS: People with MS
SCID I–IV: Structured Clinical Interview for DSM-IV Axis I Disorders
TAU: Treatment-as-usual
