# Supplementary material for: Transdiagnostic treatment of emotional disorders in people with multiple sclerosis: randomized controlled trial
Source: BMC Psychol. 2020 Oct 31;8:114. doi: 10.1186/s40359-020-00480-8 (PMC7603744; doi:10.1186/s40359-020-00480-8)
Supplement: Supplementary file 2 — Additional file 2. Treatment Protocol. [file 40359_2020_480_MOESM2_ESM.docx]

**APPENDIX 1**

**Reference of the Unified Protocol intervention:**

*David H. Barlow and Todd J. Farchione (2018) Applications of the Unified Protocol for Transdiagnostic Treatment of Emotional Disorders.* *Published in the United States of America by Oxford University Press. 198 Madison Avenue, New York, NY 10016, United States of America. © Oxford University Press 2018.*

*David H. Barlow, Todd J. Farchione, Shannon Sauer- Zavala, Heather Murray Latin, Kristen K. Ellard, Jacqueline R. Bullis, Kate H. Bentley, Hannah T Boettcher, Clair Cassiello- Robbins (2018) Unified Protocol for Transdiagnostic Treatment of Emotional Disorders Therapist Guide. Second Edition © Oxford University Press 2018, First Edition published in 2011, Second Edition published in 2018 New York, NY 10016, United States of America.*

**APPENDIX 2**

**EIGHT MODULES TECHNIQUES**

**Module 1**: The purpose of this module is to maximize the patient’s readiness for change and increase motivation to engage in treatment. It was designed to help patients clarify their goals for treatment and to explore the benefits and costs of changing and remaining the same. This module provides two exercises for enhancing the motivation necessary in initiating this type of treatment program and can be reviewed as needed throughout the course of treatment.

**Module 2**: this module provides psychoeducation on the functional, adaptive nature of emotions and assists patients in developing greater awareness of patterns of emotional responding, including potential maintaining factors (e.g., common triggers, environmental contingencies, and/ or the maintaining role of avoidance). Patients will also learn how to monitor and track their emotions by focusing on three core components of their emotional experiences (thoughts, physical feelings, and behaviors).

**Module 3:** The purpose of Module 3 is to introduce patients to cultivating a nonjudgmental, present- focused stance toward their emotional experiences. The previous module asked patients to monitor to how their emotions unfold over time. This module builds upon that work by encouraging them to incorporate mindful awareness that moves beyond simply paying attention to these experiences. The principles of mindfulness are very consistent with the overall goal of the UP— to develop a more, open, approach- oriented relationship with emotions.

**Module 4**: Module 4 focuses on one very important component of emotional experiences: thoughts. Specifically, you will work with your patient to develop a greater awareness of how their thoughts (or interpretations) influence their emotions, to learn to identify their negative thinking patterns, and to increase flexibility in interpreting different situations. The concepts introduced in this chapter are aimed to facilitate patients’ ability to approach emotion- provoking situations and respond to their emotions in more helpful, adaptive ways.

**Module 5**: focuses on the behavioral component of the emotional response and begins by reviewing the role of *emotional behaviors* (i.e., behaviors that are used to control strong emotions) in the development and maintenance of maladaptive emotional responding. In this module, you will help your patient identify relevant emotional behaviors and then work with them to develop and engage in *Alternative Actions*. Over time, it is expected that these *Alternative Actions* will help remediate cognitive and behavioral patterns contributing to the frequent occurrence of strong negative affect and maintaining your patient’s distress in response to their experience of strong emotions.

**Module 6**: This module focuses on *interoceptive exposure,* or exposure to physical sensations that are associated with (and can sometimes trigger) intense emotions. The exercises described in the UP workbook are designed to assist patients in gaining further awareness of physical sensations that are part of an emotional reaction. Further, these exercises, and continued exposures focused on physical sensations, are expected to help patients learn to tolerate and think differently about the sensations and provide an opportunity to break the conditioned association between the sensations and strong emotions such as fear, anxiety, and sadness.

**Module 7**: The primary focus of this module is *Emotion Exposures*. These are exercises designed specifically to provoke strong emotional responses so that patients can put into practice the skills they have developed thus far in therapy. Following a brief introduction to the concept of *Emotion* *Exposures* and the rationale for engaging in these exercises, you will assist your patient to gradually confront internal and external stimuli that produce intense emotional reactions while helping them modify their responses to those emotions. You will help your patient incorporate the skills learned in therapy (e.g., present- focused awareness, nonjudgment, cognitive reappraisal) into their exposure practice and address any emotion avoidance (or other behaviors) that may impede treatment progress.

Module 8: The purpose of this module is to evaluate the patient’s progress and to plan for the future. You will also reinforce the skills learned in treatment, review key treatment concepts, and help patients develop strategies for preventing “relapse.” Additionally, this chapter is used to address symptom recurrence and how patients can maintain treatment gains in the long term.

The contents and techniques include:

1. Unified model of psychopathology; motivation enhancement strategies; Treatment goal setting, decisional balance exercise, changing versus staying
2. Psychoeducation on the adaptive function of emotions; three-components the model of emotional experiences describes the sequence of events around emotions. anchoring in the present
3. Review primary emotions, Natural course of emotions and role of avoidance; present-focused, nonjudgmental emotion awareness, recognize the interaction between their thoughts, feelings, and behaviors during an emotional experience
4. Flexible thinking, automatic appraisals, thinking traps, Distress tolerance skills, familiarizing with various emotional avoidance strategies, their impact on emotional experience, and knowledge of the contradictory effects of avoiding emotions
5. Examining EDBs, knowledge, and identification of their impact on emotional experiences, identifying maladaptive EDBs, and creating alternatives for acting through behaviors
6. Increase patients’ awareness and tolerance of somatic sensations, Knowledge, and tolerance of physical senses, increase awareness of the role of emotional feelings in emotional experiences, practice exercises or visceral confrontation in order to be aware of physical sensations and increase tolerance of these symptoms
7. Emphasizes the practice of treatment concepts through in- session and out- of- session exposures to emotion experiences, focus on provoking the emotion, replace interpretations about the dangerousness of situations with more adaptive appraisals, extinguish anxious reactions to intense emotional experiences, modify EDBs, visceral confrontation, providing the opportunity for skills rehearsal and consolidation in the context of a strong emotion
8. Overview of significant treatment concepts and the patient’s progress is reviewed. Specific strategies for preserving and extending treatment gains are discussed. Prevention of recurrence, an overview of treatment concepts and discussion about patient’s healing and progress and plans for future practices

**Fidelity**

The UP treatment sessions were developed by a Ph.D. student with three years' experience in group therapy, conducted a trial with PwMS, with certification on CBT procedures and techniques and the UP***,*** under the supervision of two well-experienced associate professors. Two neurologists investigated the primary eligibility criteria related to a medical condition. The baseline interviews were conducted by a pre-doctoral student of psychology pre-doctoral, and Ph.D. students with more than three years' experience in clinical settings. The post-treatment interviews were conducted by three professional psychologists with over eight years' experience in clinical settings. Regarding safety, the medical health care staff included two physicians and four experienced nurses also alerted in case of emergency conditions during all activities. Before recruitment, all clinical team members received a comprehensive refresh course in the context of psychological assessments*,* structured interview, ethics in clinical research, MS nature, and safety anticipations and professional considerations, particularly in MS. Staff in the study sites were trained initially, and therapy supervision was provided by weekly meetings between therapists and investigators
